# 168. Safety, Reactogenicity, and Immunogenicity of mRNA-1345, an Investigational Respiratory Syncytial Virus Vaccine, in Participants Aged 2 to < 18 Years at High Risk of Severe Disease

**DOI:** 10.1093/ofid/ofae631.005

**Published:** 2025-01-29

**Authors:** Sabine Schnyder Ghamloush, Hui Qian, HuiLing Chen, Joseph Whitten, Sonia K Stoszek, Matthew D Snape

**Affiliations:** Moderna, Inc., Cambridge, MA; Moderna, Inc., Cambridge, MA; Moderna, Inc., Cambridge, MA; Moderna, Inc., Cambridge, MA; Moderna, Inc., Cambridge, MA; Moderna Biotech UK, Inc, Didcot, England, United Kingdom

## Abstract

**Background:**

Respiratory syncytial virus (RSV) is the primary cause of lower respiratory tract disease in children aged < 5 years and causes substantial burden in those at high risk aged 5-18 years. No active RSV vaccine is currently available for children. Here, we present interim safety and immunogenicity of a single injection of the mRNA-based vaccine, mRNA-1345, in participants aged 2 to < 18 years at high risk for severe RSV disease.

**Figure:**
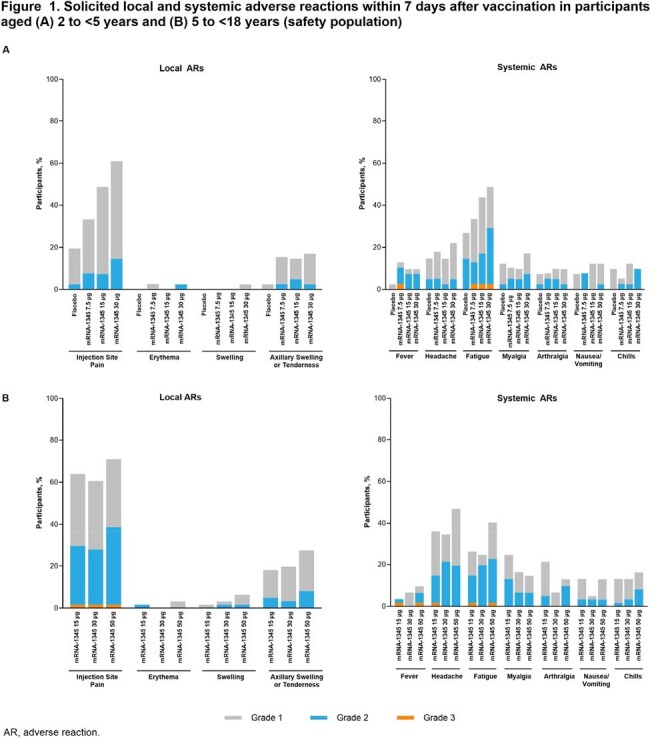

**Methods:**

This phase 2, randomized, observer-blind study enrolled participants into 2 age cohorts (2 to < 5 years and 5 to < 18 years). Participants aged 2 to < 5 years were healthy or had stable, chronic conditions increasing risk for RSV disease, and were randomized (1:1:1:1) to receive a single mRNA-1345 injection (7.5, 15, or 30 μg) or placebo. Participants aged 5 to < 18 years had chronic conditions that increased disease risk and were randomized (1:1:1) to receive a single mRNA-1345 injection (15, 30, or 50 μg). Safety and reactogenicity were primary objectives. The secondary objective was immunogenicity, measured by serum RSV neutralizing antibodies (nAb) against RSV-A and RSV-B, and RSV prefusion F (preF)-binding antibodies (bAb). Interim results through Day 29 are reported.

**Figure:**
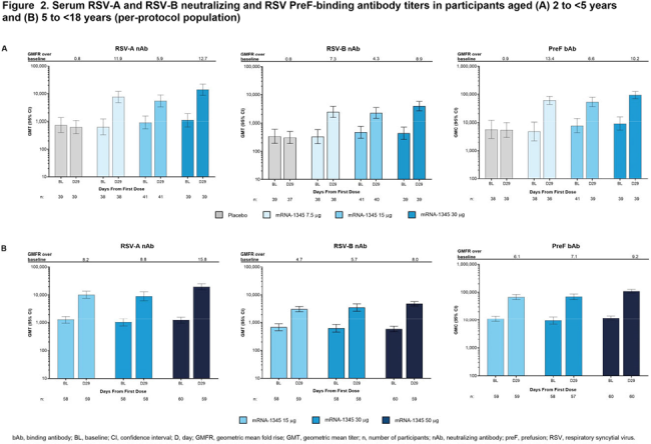

**Results:**

At data cutoff, 162 participants aged 2 to < 5 years (mean 2.9 ± 0.8 years) and 184 participants aged 5 to < 18 years (mean 11.4 ± 3.8 years) had received a vaccination. In both cohorts, solicited adverse reactions were primarily Grade 1-2 in severity; injection site pain, headache, and fatigue were most frequently reported (**Figure 1**). No deaths occurred and no adverse events (AEs) led to study discontinuation; 1 unrelated serious AE and 1 AE of special interest were reported. At Day 29, a single mRNA-1345 injection increased RSV nAb titers from baseline by 5.9- to 12.7-fold (RSV-A) and 4.3- to 8.9-fold (RSV-B) in those aged 2 to < 5 years, and by 8.2- to 15.8-fold (RSV-A) and 4.7- to 8.0-fold (RSV-B) in those aged 5 to < 18 years (**Figure 2**). Similarly, vaccination increased RSV preF bAb concentrations at Day 29 for both cohorts (**Figure 2**).

**Conclusion:**

A single injection of mRNA-1345 was well tolerated, raised no safety concerns, and increased both nAb and bAb immune responses in participants aged 2 to < 18 years. These findings support further development of mRNA-1345 for the pediatric population.

**Disclosures:**

**Sabine Schnyder Ghamloush, MD**, Moderna, Inc.: Employee|Moderna, Inc.: Stocks/Bonds (Public Company) **Hui Qian, PhD**, Moderna, Inc.: Employee|Moderna, Inc.: Stocks/Bonds (Public Company) **HuiLing Chen, PhD**, Moderna, Inc.: Employee|Moderna, Inc.: Stocks/Bonds (Public Company) **Joseph Whitten, MD**, Moderna, Inc.: Employee|Moderna, Inc.: Stocks/Bonds (Public Company) **Sonia K. Stoszek, PhD**, Moderna, Inc.: Employee|Moderna, Inc.: Stocks/Bonds (Public Company) **Matthew D. Snape, MBBS, MD, FRCPCH, FPM, FMedSci**, Moderna, Inc.: Emplolyee|Moderna, Inc.: Stocks/Bonds (Public Company)

